# Crystal structure of (*E*)-2-{[(6-meth­oxy-1,3-benzo­thia­zol-2-yl)imino]­meth­yl}phenol

**DOI:** 10.1107/S2056989015005228

**Published:** 2015-03-21

**Authors:** Yousef Hijji, Belygona Barare, Gilbert Wairia, Ray J. Butcher, Jan Wikaira

**Affiliations:** a Department of Chemistry and Earth Sciences, Qatar University, Doha, Qatar; b Chemistry Department, Morgan State University, Baltimore, MD 21251, USA; c Department of Chemistry, Howard University, 525 College Street NW, Washington, DC 20059, USA; d Department of Chemistry, University of Canterbury, Private Bag 4800, Christchurch, New Zealand

**Keywords:** crystal structure, amino­benzo­thia­zole derivatives, amino­thia­zole Schiff bases, hydrogen bonding

## Abstract

The title compound, C_15_H_12_N_2_O_2_S, crystallizes in the ortho­rhom­bic space group *Pna2_1_*, with two mol­ecules in the asymmetric unit (*Z*′ = 2). Each mol­ecule consists of a 2-hy­droxy Schiff base moiety linked through a spacer to a 2-amino­benzo­thia­zole moiety. Each mol­ecule contains an intra­molecular hydrogen bond between the –OH group and imine N atom, forming a six-membered ring. The two independent molecules are linked by a pair of C—H⋯O hydrogen bonds, forming dimers with an *R* ^2^_2_(20) ring motif. These dimers are further lined into sheets in the *ab* plane by weak inter­molecular C—H⋯N inter­actions. The structure was refined as an inversion twin

## Abstract 2

The title compound crystallizes with two mol­ecules in the asymmetric unit (*Z*′ = 2) which are linked into dimers by 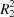
(20) C—H⋯O inter­actions. These dimers are further linked into sheets in the *ab* plane by weak inter­molecular C—H⋯N inter­actions.

## Chemical context   

A wide range of biological activities have been attributed to amino­thia­zoles and compounds having similar structures (Tahiliani *et al.*, 2003 ▸) and they have many applications in both human and veterinary medicine (Smith *et al.*, 1999 ▸; Sarhan *et al.*, 2010 ▸). Certain 2-amino­benzo­thia­zole derivatives act on the central nervous system (Funderburk *et al.*, 1953 ▸), possess anti­microbial (Murhekar & Khadsan, 2010 ▸; Ravi *et al.*, 2014 ▸), anti­fungal (Catalano *et al.*, 2013 ▸) and anti­bacterial properties (Asiri *et al.*, 2013 ▸), serve as selective receptors for anion sensing (Hijji & Wairia, 2005 ▸), are active in corrosion inhibition (Quraishi *et al.*, 1997 ▸; Rawat & Quraishi, 2003 ▸) and act as plant-growth regulators (Mahajan *et al.*, 2013 ▸). In addition, some metal complexes of Schiff bases of 2-amino­benzo­thia­zole derivatives have potent anti­bacterial properties (Sharma *et al.*, 2002 ▸; Song *et al.*, 2010 ▸). Among anti­tumor agents discovered in recent years, the identification of various 2-(4-amino­phen­yl)benzo­thia­zoles as potent and selective anti­tumor drugs against breast, ovarian, colon and renal cell lines has stimulated remarkable inter­est (Usman *et al.*, 2003 ▸; Shi *et al.*, 1996 ▸; Havrylyuk *et al.* 2010 ▸) in this class of compound from both a synthetic, and particularly, a structural point of view. Amino­thia­zole Schiff bases have been prepared as inter­mediate ligands and for complexation with various metals (Liang *et al.*,1999 ▸; Liu *et al.*, 2009 ▸).
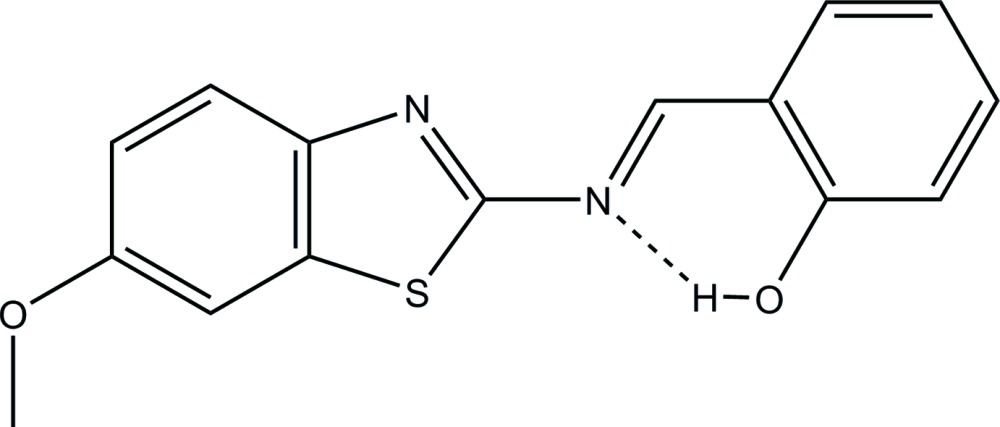


 In this context, the synthesis and structural characterization of new 2-amino­benzo­thia­zole Schiff base derivatives is of inter­est (El’tsov & Mokrushin, 2002 ▸).

## Structural commentary   

The title compound, C_15_H_12_N_2_O_2_S, crystallizes in the ortho­rhom­bic space group, *Pna2_1_*, with two mol­ecules (*A* and *B*) in the asymmetric unit (*Z*′ = 2). Each mol­ecule consists of a 2-hy­droxy Schiff base moiety linked through a spacer to a 2-amino­benzo­thia­zole moiety. This spacer is both planar [r.m.s. deviations of fitted atoms of 0.004 (3) and 0.007 (3) Å, respectively for mol­ecules *A* and *B*] and very close to coplanar with both the Schiff base and 2-amino­benzo­thia­zole end moieties [making dihedral angles of 2.6 (9) and 4.0 (3)°, respectively, in mol­ecule *A* and 3.3 (8) and 3.9 (7)° in mol­ecule *B*]. The mol­ecules themselves are very close to planar, as is shown by the dihedral angles of 4.0 (3) and 6.3 (2) between the two end groups for mol­ecules *A* and *B*, respectively. Each mol­ecule contains an intra­molecular hydrogen bond between the OH group and imine N atom, forming a six-membered ring.

## Supra­molecular features   

**Figure 1:**
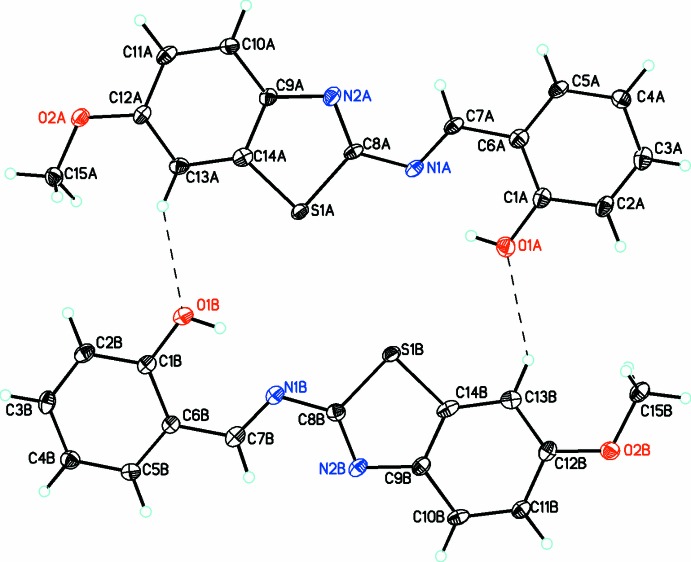
Mol­ecular diagram for mol­ecules *A* and *B* of the title compound, showing the atom labeling. Displacement parameters are drawn at the 30% probability level. The diagram shows the two mol­ecules (*A* and *B*) linked into dimers by 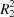
(20) C—H⋯O hydrogen bonds (dashed lines; see Table 1 ▸ for details).

**Figure 2:**
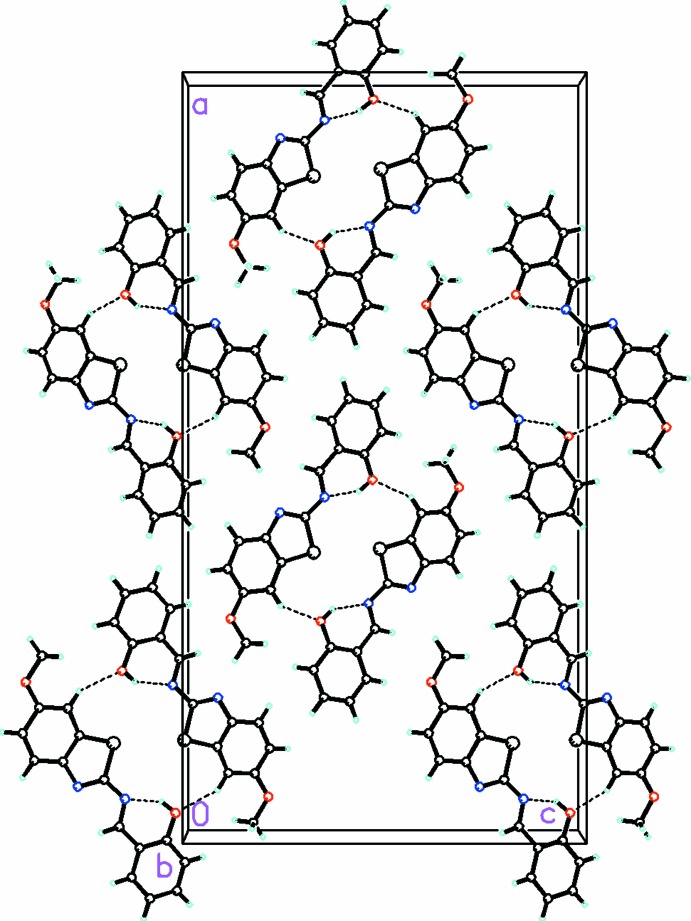
Packing diagram, viewed along the *b* axis, showing a sheet of 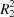
(20) C—H⋯O-linked dimers in the *ac* plane.

In addition to the intra­molecular hydrogen bond mentioned above, the mol­ecules are linked by a pair of C—H⋯O hydrogen bonds (Table 1 ▸), forming dimers with an 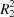
(20) ring motif, as shown in Fig. 1 ▸. These dimers are further linked into sheets in the *ab* plane by weak inter­molecular C—H⋯N inter­actions involving C15 and N2*B*, as shown in Fig. 2 ▸.

## Database survey   

A search of the Cambridge Structural Database (CSD, Version 5.35, last update November 2014; Groom & Allen, 2014 ▸) for related Schiff base derivatives of 2-amino­benzo­thia­zole gave 23 hits of which the closest example to the title compound was (*E*)-2-[(6-eth­oxy­benzo­thia­zol-2-yl)imino­meth­yl]-6-meth­oxy­phenol (Kong, 2009 ▸).

## Synthesis and crystallization   

A mixture of 0.505 g (4.10 mmol) salicyl­aldehyde and 0.746 g (4.10 mmol) 2-amino-6-meth­oxy­benzo­thio­zole was dissolved in 2 ml of aceto­nitrile in a vial. The mixture was reacted in a Biotage initiator eight mono mode microwave at 423 K for 2 min and then allowed to cool for 15 min. The resulting product was recrystallized from aceto­nitrile, filtered and then vacuum dried to afford 0.971 g (86% yield) of a yellow crystalline solid (m.p. 399–403 K). A sample was dissolved in ethanol and allowed to crystallize by slow evaporation to give yellow needles used for X-ray structural determination.

^1^H NMR (300 MHz, CDCl_3_): δ 12.07 (*s*, 1H), 9.36 (*s*, 1H), 8.81 (*dd*, *J* = 9.0, 2.5 Hz, 1H), 8.39 (*d*, *J* = 7.5 Hz, 1H), 8.05 (*d*, *J* = 9.0 Hz. 1H), 7.55 (*m*, 2H), 7.09 (*d*, 7.5 Hz, 1H), 7.04 (*t*, *J* = 7.5 Hz, 1H), 3.83 (*s*, 3H)

^13^C NMR (300 MHz, CDCl_3_, p.p.m.): δ 55.07, 105.07, 115.46, 118.4, 121.2, 122.88, 125.26, 130.4, 132.44, 135.07, 145.59, 157.8 162.69, 165.36, 169.49

## Refinement   

Crystal data, data collection and structure refinement details are summarized in Table 2 ▸. C-bound H atoms were positioned geometrically and refined as riding: C–H = 0.93–0.99 Å with *U_iso_*(H) = 1.5*U*_eq_(C) for methyl H atoms and = 1.2*U_eq_*(C) for other H atoms. Phenol H atoms were located in a difference Fourier map and then refined as riding on their attached O atoms.

## Supplementary Material

Crystal structure: contains datablock(s) I. DOI: 10.1107/S2056989015005228/hg5435sup1.cif

Structure factors: contains datablock(s) I. DOI: 10.1107/S2056989015005228/hg5435Isup2.hkl

Supporting information file. DOI: 10.1107/S2056989015005228/hg5435Isup3.cml

CCDC reference: 1053989

Additional supporting information:  crystallographic information; 3D view; checkCIF report

## Figures and Tables

**Table 1:** Hydrogen-bond geometry (, )

*D*H*A*	*D*H	H*A*	*D* *A*	*D*H*A*
O1*A*H1*A*N1*A*	0.84	1.93	2.647(9)	143
C13*A*H13*A*O1*B*	0.95	2.48	3.289(9)	144
C15*A*H15*A*N2*B* ^i^	0.98	2.57	3.525(10)	166
O1*B*H1*B*N1*B*	0.84	1.89	2.636(9)	147
C13*B*H13*B*O1*A*	0.95	2.53	3.356(10)	145

Symmetry code: (i) 
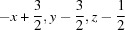
.

**Table 2:** Experimental details

Crystal data	
Chemical formula	C_15_H_12_N_2_O_2_S
*M*_r_	284.33
Crystal system, space group	Orthorhombic, *P* *n* *a*2_1_
Temperature (K)	120
*a*, *b*, *c* ()	35.623(2), 3.8172(2), 18.6525(8)
*V* (^3^)	2536.4(2)
*Z*	8
Radiation type	Cu *K*
(mm^1^)	2.30
Crystal size (mm)	0.38 0.09 0.06

Data collection	
Diffractometer	Agilent SuperNova (Dual, Cu at zero, Atlas)
Absorption correction	Multi-scan (*CrysAlis PRO*; Agilent, 2012 ▸)
*T*_min_, *T*_max_	0.573, 0.863
No. of measured, independent and observed [*I* > 2(*I*)] reflections	6990, 3895, 3677
*R*_int_	0.045
(sin /)_max_ (^1^)	0.630

Refinement	
*R*[*F* ^2^ > 2(*F* ^2^)], *wR*(*F* ^2^), *S*	0.073, 0.189, 1.09
No. of reflections	3895
No. of parameters	364
No. of restraints	1
H-atom treatment	H-atom parameters constrained
_max_, _min_ (e ^3^)	1.01, 0.74
Absolute structure	Refined as an inversion twin
Absolute structure parameter	0.65(5)

Computer programs: *CrysAlis PRO* (Agilent, 2012 ▸), *SUPERFLIP* (Palatinus *et al.*, 2007 ▸), *SHELXL2013* (Sheldrick, 2015 ▸) and *SHELXTL* (Sheldrick, 2008 ▸).

## supplementary crystallographic information

### Crystal data

**Table:**

C_15_H_12_N_2_O_2_S	*D*_x_ = 1.489 Mg m^−^^3^
*M**_r_* = 284.33	Cu *K*α radiation, λ = 1.54178 Å
Orthorhombic, *P**n**a*2_1_	Cell parameters from 2917 reflections
*a* = 35.623 (2) Å	θ = 4.7–76.1°
*b* = 3.8172 (2) Å	µ = 2.30 mm^−^^1^
*c* = 18.6525 (8) Å	*T* = 120 K
*V* = 2536.4 (2) Å^3^	Needle, yellow–orange
*Z* = 8	0.38 × 0.09 × 0.06 mm
*F*(000) = 1184	

### Data collection

**Table:**

Agilent SuperNova (Dual, Cu at zero, Atlas) diffractometer	3895 independent reflections
Radiation source: sealed X-ray tube	3677 reflections with *I* > 2σ(*I*)
Detector resolution: 5.3250 pixels mm^-1^	*R*_int_ = 0.045
ω scans	θ_max_ = 76.2°, θ_min_ = 3.4°
Absorption correction: multi-scan (*CrysAlis PRO*; Agilent, 2012)	*h* = −41→44
*T*_min_ = 0.573, *T*_max_ = 0.863	*k* = −2→4
6990 measured reflections	*l* = −20→23

### Refinement

**Table:**

Refinement on *F*^2^	Hydrogen site location: mixed
Least-squares matrix: full	H-atom parameters constrained
*R*[*F*^2^ > 2σ(*F*^2^)] = 0.073	*w* = 1/[σ^2^(*F*_o_^2^) + (0.0845*P*)^2^ + 6.6687*P*] where *P* = (*F*_o_^2^ + 2*F*_c_^2^)/3
*wR*(*F*^2^) = 0.189	(Δ/σ)_max_ < 0.001
*S* = 1.09	Δρ_max_ = 1.01 e Å^−^^3^
3895 reflections	Δρ_min_ = −0.74 e Å^−^^3^
364 parameters	Absolute structure: Refined as an inversion twin.
1 restraint	Absolute structure parameter: 0.65 (5)

### Special details

**Table:**

Geometry. All e.s.d.'s (except the e.s.d. in the dihedral angle between two l.s. planes) are estimated using the full covariance matrix. The cell e.s.d.'s are taken into account individually in the estimation of e.s.d.'s in distances, angles and torsion angles; correlations between e.s.d.'s in cell parameters are only used when they are defined by crystal symmetry. An approximate (isotropic) treatment of cell e.s.d.'s is used for estimating e.s.d.'s involving l.s. planes.
Refinement. Refined as a 2-component inversion twin.

### Fractional atomic coordinates and isotropic or equivalent isotropic displacement parameters (Å^2^)

**Table:**

	*x*	*y*	*z*	*U*_iso_*/*U*_eq_	
S1A	0.87402 (5)	0.3471 (4)	0.32176 (9)	0.0241 (4)	
O1A	0.97069 (15)	0.6508 (16)	0.4723 (3)	0.0337 (13)	
H1A	0.9559	0.6313	0.4376	0.050*	
O2A	0.78912 (15)	−0.1334 (15)	0.1150 (3)	0.0283 (12)	
N1A	0.94790 (18)	0.3743 (16)	0.3497 (4)	0.0250 (13)	
N2A	0.92781 (17)	0.1374 (18)	0.2372 (4)	0.0283 (13)	
C1A	1.0055 (2)	0.5657 (19)	0.4512 (4)	0.0261 (15)	
C2A	1.0359 (2)	0.648 (2)	0.4958 (4)	0.0306 (16)	
H1	1.0314	0.7620	0.5402	0.037*	
C3A	1.0718 (2)	0.568 (2)	0.4768 (4)	0.0318 (17)	
H2	1.0919	0.6241	0.5082	0.038*	
C4A	1.0793 (2)	0.403 (2)	0.4114 (5)	0.0274 (15)	
H3	1.1045	0.3592	0.3970	0.033*	
C5A	1.0495 (2)	0.303 (2)	0.3674 (4)	0.0274 (16)	
H4	1.0542	0.1730	0.3249	0.033*	
C6A	1.0126 (2)	0.394 (2)	0.3862 (4)	0.0274 (16)	
C7A	0.9824 (2)	0.2951 (18)	0.3370 (4)	0.0238 (15)	
H5	0.9884	0.1694	0.2946	0.029*	
C8A	0.9213 (2)	0.2730 (17)	0.2996 (4)	0.0225 (14)	
C9A	0.89431 (19)	0.0635 (19)	0.2017 (4)	0.0237 (14)	
C10A	0.8905 (2)	−0.087 (2)	0.1348 (4)	0.0265 (15)	
H10A	0.9120	−0.1510	0.1078	0.032*	
C11A	0.8548 (2)	−0.1449 (19)	0.1075 (4)	0.0264 (16)	
H11A	0.8521	−0.2508	0.0617	0.032*	
C12A	0.8227 (2)	−0.0498 (17)	0.1462 (4)	0.0212 (14)	
C13A	0.8255 (2)	0.1077 (19)	0.2129 (4)	0.0254 (15)	
H13A	0.8037	0.1706	0.2392	0.031*	
C14A	0.8617 (2)	0.1715 (18)	0.2403 (4)	0.0240 (14)	
C15A	0.75623 (19)	−0.027 (2)	0.1521 (5)	0.0289 (16)	
H15A	0.7340	−0.1033	0.1254	0.043*	
H15B	0.7560	0.2289	0.1566	0.043*	
H15C	0.7560	−0.1328	0.2000	0.043*	
S1B	0.87656 (5)	0.8509 (4)	0.49182 (9)	0.0246 (4)	
O1B	0.78077 (16)	0.5412 (16)	0.3390 (3)	0.0361 (14)	
H1B	0.7960	0.6119	0.3701	0.054*	
O2B	0.95964 (15)	1.3166 (15)	0.7026 (3)	0.0293 (12)	
N1B	0.80272 (18)	0.8089 (15)	0.4624 (4)	0.0247 (13)	
N2B	0.82255 (17)	1.0626 (16)	0.5746 (4)	0.0263 (13)	
C1B	0.7453 (2)	0.5753 (18)	0.3644 (4)	0.0248 (15)	
C2B	0.7160 (2)	0.4583 (19)	0.3210 (5)	0.0291 (15)	
H6	0.7209	0.3632	0.2749	0.035*	
C3B	0.6790 (2)	0.484 (2)	0.3472 (4)	0.0299 (17)	
H7	0.6587	0.4020	0.3187	0.036*	
C4B	0.6717 (2)	0.628 (2)	0.4136 (4)	0.0287 (16)	
H8	0.6466	0.6431	0.4301	0.034*	
C5B	0.7005 (2)	0.7494 (18)	0.4564 (4)	0.0243 (15)	
H9	0.6950	0.8494	0.5019	0.029*	
C6B	0.7381 (2)	0.7258 (16)	0.4329 (4)	0.0204 (14)	
C7B	0.7681 (2)	0.8352 (17)	0.4793 (4)	0.0245 (15)	
H10	0.7618	0.9319	0.5247	0.029*	
C8B	0.8289 (2)	0.9165 (19)	0.5123 (4)	0.0252 (15)	
C9B	0.8560 (2)	1.1290 (19)	0.6098 (4)	0.0253 (15)	
C10B	0.8589 (2)	1.2798 (19)	0.6777 (4)	0.0262 (16)	
H10B	0.8370	1.3415	0.7040	0.031*	
C11B	0.8943 (2)	1.3379 (19)	0.7062 (5)	0.0261 (15)	
H11B	0.8967	1.4428	0.7522	0.031*	
C12B	0.9269 (2)	1.2432 (18)	0.6678 (5)	0.0252 (16)	
C13B	0.9242 (2)	1.0870 (17)	0.6009 (4)	0.0246 (14)	
H13B	0.9460	1.0207	0.5748	0.029*	
C14B	0.8884 (2)	1.0313 (17)	0.5736 (4)	0.0234 (14)	
C15B	0.9936 (2)	1.235 (2)	0.6643 (5)	0.0288 (16)	
H15D	1.0149	1.3469	0.6882	0.043*	
H15E	0.9916	1.3226	0.6150	0.043*	
H15F	0.9972	0.9808	0.6635	0.043*	

### Atomic displacement parameters (Å^2^)

**Table:**

	*U*^11^	*U*^22^	*U*^33^	*U*^12^	*U*^13^	*U*^23^
S1A	0.0280 (8)	0.0261 (8)	0.0182 (9)	−0.0016 (6)	−0.0003 (7)	−0.0049 (7)
O1A	0.029 (3)	0.044 (3)	0.028 (3)	−0.005 (2)	0.000 (2)	0.001 (3)
O2A	0.031 (3)	0.031 (3)	0.023 (3)	−0.003 (2)	−0.002 (2)	−0.004 (2)
N1A	0.032 (3)	0.021 (3)	0.022 (3)	−0.002 (2)	−0.002 (3)	−0.006 (2)
N2A	0.032 (3)	0.033 (3)	0.020 (3)	−0.007 (2)	−0.003 (3)	0.001 (3)
C1A	0.033 (4)	0.025 (3)	0.020 (4)	−0.006 (3)	−0.003 (3)	0.006 (3)
C2A	0.040 (4)	0.034 (4)	0.018 (4)	−0.015 (3)	−0.002 (3)	0.002 (3)
C3A	0.039 (4)	0.033 (4)	0.024 (4)	−0.007 (3)	−0.008 (3)	0.009 (3)
C4A	0.027 (3)	0.026 (3)	0.030 (4)	0.004 (3)	0.001 (3)	0.002 (3)
C5A	0.038 (4)	0.030 (4)	0.014 (4)	−0.001 (3)	−0.001 (3)	0.000 (3)
C6A	0.034 (4)	0.025 (3)	0.023 (4)	−0.002 (3)	0.000 (3)	0.004 (3)
C7A	0.037 (4)	0.022 (3)	0.012 (3)	−0.003 (3)	0.000 (3)	0.003 (3)
C8A	0.034 (4)	0.017 (3)	0.017 (4)	0.000 (2)	0.002 (3)	−0.004 (2)
C9A	0.028 (3)	0.027 (3)	0.016 (3)	−0.004 (3)	0.001 (3)	0.007 (3)
C10A	0.030 (3)	0.028 (4)	0.022 (4)	0.003 (3)	0.003 (3)	−0.002 (3)
C11A	0.041 (4)	0.021 (3)	0.016 (4)	−0.008 (3)	0.000 (3)	0.003 (3)
C12A	0.032 (3)	0.014 (3)	0.018 (3)	−0.004 (2)	−0.003 (3)	0.004 (3)
C13A	0.030 (3)	0.027 (4)	0.019 (3)	0.002 (3)	0.004 (3)	0.004 (3)
C14A	0.033 (3)	0.016 (3)	0.023 (4)	−0.005 (3)	0.003 (3)	0.005 (3)
C15A	0.025 (3)	0.029 (3)	0.033 (4)	−0.003 (3)	−0.002 (3)	−0.005 (3)
S1B	0.0291 (9)	0.0271 (8)	0.0177 (9)	0.0008 (6)	−0.0003 (6)	−0.0047 (7)
O1B	0.033 (3)	0.046 (3)	0.029 (3)	−0.001 (2)	0.002 (2)	−0.013 (3)
O2B	0.029 (3)	0.036 (3)	0.022 (3)	0.004 (2)	−0.001 (2)	−0.001 (2)
N1B	0.032 (3)	0.020 (3)	0.022 (3)	0.002 (2)	−0.004 (3)	0.002 (2)
N2B	0.034 (3)	0.020 (3)	0.024 (3)	−0.002 (2)	−0.004 (3)	−0.003 (3)
C1B	0.033 (4)	0.018 (3)	0.023 (4)	0.001 (3)	−0.003 (3)	0.002 (3)
C2B	0.040 (4)	0.029 (3)	0.018 (4)	0.000 (3)	0.000 (3)	0.003 (3)
C3B	0.036 (4)	0.028 (4)	0.026 (4)	−0.002 (3)	−0.010 (3)	0.009 (3)
C4B	0.030 (4)	0.030 (4)	0.026 (4)	0.000 (3)	0.003 (3)	0.002 (3)
C5B	0.031 (4)	0.020 (3)	0.022 (4)	0.001 (3)	0.003 (3)	0.003 (3)
C6B	0.029 (3)	0.012 (3)	0.021 (4)	0.001 (2)	−0.002 (3)	0.004 (3)
C7B	0.040 (4)	0.014 (3)	0.019 (4)	−0.003 (2)	−0.003 (3)	0.009 (3)
C8B	0.028 (4)	0.022 (3)	0.025 (4)	0.002 (3)	0.001 (3)	0.000 (3)
C9B	0.033 (3)	0.019 (3)	0.024 (4)	−0.004 (2)	−0.001 (3)	−0.001 (3)
C10B	0.040 (4)	0.017 (3)	0.021 (4)	0.001 (3)	0.005 (3)	−0.002 (3)
C11B	0.033 (4)	0.021 (3)	0.024 (4)	−0.002 (3)	0.000 (3)	−0.002 (3)
C12B	0.033 (4)	0.014 (3)	0.028 (4)	−0.004 (2)	−0.003 (3)	0.002 (3)
C13B	0.033 (3)	0.018 (3)	0.023 (4)	0.002 (3)	0.003 (3)	0.006 (3)
C14B	0.045 (4)	0.014 (3)	0.011 (3)	0.000 (3)	0.003 (3)	0.006 (2)
C15B	0.040 (4)	0.025 (3)	0.021 (4)	−0.002 (3)	−0.004 (3)	0.001 (3)

### Geometric parameters (Å, º)

**Table:**

S1A—C14A	1.718 (8)	S1B—C14B	1.726 (8)
S1A—C8A	1.759 (8)	S1B—C8B	1.758 (8)
O1A—C1A	1.341 (10)	O1B—C1B	1.356 (9)
O1A—H1A	0.8399	O1B—H1B	0.8400
O2A—C12A	1.369 (9)	O2B—C12B	1.364 (9)
O2A—C15A	1.421 (9)	O2B—C15B	1.438 (10)
N1A—C7A	1.288 (10)	N1B—C7B	1.278 (10)
N1A—C8A	1.385 (10)	N1B—C8B	1.380 (10)
N2A—C8A	1.295 (10)	N2B—C8B	1.309 (10)
N2A—C9A	1.393 (9)	N2B—C9B	1.383 (9)
C1A—C6A	1.402 (11)	C1B—C2B	1.395 (11)
C1A—C2A	1.402 (11)	C1B—C6B	1.424 (10)
C2A—C3A	1.362 (12)	C2B—C3B	1.409 (11)
C2A—H1	0.9500	C2B—H6	0.9500
C3A—C4A	1.398 (12)	C3B—C4B	1.378 (12)
C3A—H2	0.9500	C3B—H7	0.9500
C4A—C5A	1.395 (11)	C4B—C5B	1.381 (11)
C4A—H3	0.9500	C4B—H8	0.9500
C5A—C6A	1.405 (12)	C5B—C6B	1.412 (10)
C5A—H4	0.9500	C5B—H9	0.9500
C6A—C7A	1.463 (11)	C6B—C7B	1.435 (10)
C7A—H5	0.9500	C7B—H10	0.9500
C9A—C10A	1.382 (11)	C9B—C14B	1.388 (11)
C9A—C14A	1.428 (10)	C9B—C10B	1.396 (11)
C10A—C11A	1.385 (11)	C10B—C11B	1.388 (12)
C10A—H10A	0.9500	C10B—H10B	0.9500
C11A—C12A	1.401 (11)	C11B—C12B	1.410 (11)
C11A—H11A	0.9500	C11B—H11B	0.9500
C12A—C13A	1.385 (11)	C12B—C13B	1.387 (11)
C13A—C14A	1.407 (10)	C13B—C14B	1.391 (11)
C13A—H13A	0.9500	C13B—H13B	0.9500
C15A—H15A	0.9800	C15B—H15D	0.9800
C15A—H15B	0.9800	C15B—H15E	0.9800
C15A—H15C	0.9800	C15B—H15F	0.9800
			
C14A—S1A—C8A	88.5 (4)	C14B—S1B—C8B	89.2 (4)
C1A—O1A—H1A	109.5	C1B—O1B—H1B	109.3
C12A—O2A—C15A	116.6 (6)	C12B—O2B—C15B	116.0 (6)
C7A—N1A—C8A	117.5 (6)	C7B—N1B—C8B	117.6 (7)
C8A—N2A—C9A	110.8 (6)	C8B—N2B—C9B	110.5 (6)
O1A—C1A—C6A	122.3 (7)	O1B—C1B—C2B	117.6 (7)
O1A—C1A—C2A	119.1 (7)	O1B—C1B—C6B	121.3 (6)
C6A—C1A—C2A	118.6 (7)	C2B—C1B—C6B	121.1 (7)
C3A—C2A—C1A	121.4 (8)	C1B—C2B—C3B	118.4 (8)
C3A—C2A—H1	119.3	C1B—C2B—H6	120.8
C1A—C2A—H1	119.3	C3B—C2B—H6	120.8
C2A—C3A—C4A	120.5 (7)	C4B—C3B—C2B	121.0 (7)
C2A—C3A—H2	119.8	C4B—C3B—H7	119.5
C4A—C3A—H2	119.8	C2B—C3B—H7	119.5
C5A—C4A—C3A	119.4 (7)	C3B—C4B—C5B	120.9 (7)
C5A—C4A—H3	120.3	C3B—C4B—H8	119.5
C3A—C4A—H3	120.3	C5B—C4B—H8	119.5
C4A—C5A—C6A	119.9 (7)	C4B—C5B—C6B	120.2 (7)
C4A—C5A—H4	120.1	C4B—C5B—H9	119.9
C6A—C5A—H4	120.1	C6B—C5B—H9	119.9
C1A—C6A—C5A	120.0 (7)	C5B—C6B—C1B	118.3 (7)
C1A—C6A—C7A	122.1 (7)	C5B—C6B—C7B	120.0 (7)
C5A—C6A—C7A	117.9 (7)	C1B—C6B—C7B	121.6 (7)
N1A—C7A—C6A	121.7 (7)	N1B—C7B—C6B	123.1 (7)
N1A—C7A—H5	119.2	N1B—C7B—H10	118.4
C6A—C7A—H5	119.2	C6B—C7B—H10	118.4
N2A—C8A—N1A	126.7 (7)	N2B—C8B—N1B	127.5 (7)
N2A—C8A—S1A	116.5 (6)	N2B—C8B—S1B	114.9 (6)
N1A—C8A—S1A	116.8 (5)	N1B—C8B—S1B	117.6 (6)
C10A—C9A—N2A	126.7 (7)	N2B—C9B—C14B	115.8 (7)
C10A—C9A—C14A	119.7 (7)	N2B—C9B—C10B	124.8 (7)
N2A—C9A—C14A	113.6 (7)	C14B—C9B—C10B	119.3 (7)
C9A—C10A—C11A	119.2 (7)	C11B—C10B—C9B	118.8 (8)
C9A—C10A—H10A	120.4	C11B—C10B—H10B	120.6
C11A—C10A—H10A	120.4	C9B—C10B—H10B	120.6
C10A—C11A—C12A	121.2 (8)	C10B—C11B—C12B	120.8 (8)
C10A—C11A—H11A	119.4	C10B—C11B—H11B	119.6
C12A—C11A—H11A	119.4	C12B—C11B—H11B	119.6
O2A—C12A—C13A	123.1 (7)	O2B—C12B—C13B	125.1 (7)
O2A—C12A—C11A	115.8 (7)	O2B—C12B—C11B	114.2 (7)
C13A—C12A—C11A	121.1 (7)	C13B—C12B—C11B	120.7 (7)
C12A—C13A—C14A	117.8 (7)	C12B—C13B—C14B	117.3 (7)
C12A—C13A—H13A	121.1	C12B—C13B—H13B	121.4
C14A—C13A—H13A	121.1	C14B—C13B—H13B	121.4
C13A—C14A—C9A	120.8 (7)	C9B—C14B—C13B	123.0 (7)
C13A—C14A—S1A	128.5 (6)	C9B—C14B—S1B	109.6 (6)
C9A—C14A—S1A	110.5 (6)	C13B—C14B—S1B	127.4 (6)
O2A—C15A—H15A	109.5	O2B—C15B—H15D	109.5
O2A—C15A—H15B	109.5	O2B—C15B—H15E	109.5
H15A—C15A—H15B	109.5	H15D—C15B—H15E	109.5
O2A—C15A—H15C	109.5	O2B—C15B—H15F	109.5
H15A—C15A—H15C	109.5	H15D—C15B—H15F	109.5
H15B—C15A—H15C	109.5	H15E—C15B—H15F	109.5
			
O1A—C1A—C2A—C3A	−179.9 (7)	O1B—C1B—C2B—C3B	178.6 (6)
C6A—C1A—C2A—C3A	0.7 (11)	C6B—C1B—C2B—C3B	−1.5 (11)
C1A—C2A—C3A—C4A	0.6 (12)	C1B—C2B—C3B—C4B	1.1 (11)
C2A—C3A—C4A—C5A	−3.6 (12)	C2B—C3B—C4B—C5B	−0.1 (12)
C3A—C4A—C5A—C6A	5.3 (12)	C3B—C4B—C5B—C6B	−0.6 (11)
O1A—C1A—C6A—C5A	−178.4 (7)	C4B—C5B—C6B—C1B	0.2 (10)
C2A—C1A—C6A—C5A	1.0 (11)	C4B—C5B—C6B—C7B	−176.6 (6)
O1A—C1A—C6A—C7A	−0.1 (11)	O1B—C1B—C6B—C5B	−179.3 (6)
C2A—C1A—C6A—C7A	179.3 (7)	C2B—C1B—C6B—C5B	0.9 (10)
C4A—C5A—C6A—C1A	−4.0 (11)	O1B—C1B—C6B—C7B	−2.5 (10)
C4A—C5A—C6A—C7A	177.6 (7)	C2B—C1B—C6B—C7B	177.6 (6)
C8A—N1A—C7A—C6A	179.3 (6)	C8B—N1B—C7B—C6B	−178.6 (6)
C1A—C6A—C7A—N1A	2.9 (11)	C5B—C6B—C7B—N1B	177.4 (6)
C5A—C6A—C7A—N1A	−178.8 (7)	C1B—C6B—C7B—N1B	0.7 (10)
C9A—N2A—C8A—N1A	179.1 (7)	C9B—N2B—C8B—N1B	179.2 (7)
C9A—N2A—C8A—S1A	−2.8 (8)	C9B—N2B—C8B—S1B	−0.3 (8)
C7A—N1A—C8A—N2A	−7.7 (11)	C7B—N1B—C8B—N2B	−4.1 (11)
C7A—N1A—C8A—S1A	174.3 (5)	C7B—N1B—C8B—S1B	175.5 (5)
C14A—S1A—C8A—N2A	1.2 (6)	C14B—S1B—C8B—N2B	0.4 (6)
C14A—S1A—C8A—N1A	179.5 (6)	C14B—S1B—C8B—N1B	−179.2 (6)
C8A—N2A—C9A—C10A	−178.8 (7)	C8B—N2B—C9B—C14B	0.0 (9)
C8A—N2A—C9A—C14A	3.3 (9)	C8B—N2B—C9B—C10B	−179.3 (7)
N2A—C9A—C10A—C11A	179.6 (7)	N2B—C9B—C10B—C11B	−178.8 (7)
C14A—C9A—C10A—C11A	−2.7 (11)	C14B—C9B—C10B—C11B	2.0 (11)
C9A—C10A—C11A—C12A	0.6 (11)	C9B—C10B—C11B—C12B	−0.7 (11)
C15A—O2A—C12A—C13A	4.5 (10)	C15B—O2B—C12B—C13B	2.6 (10)
C15A—O2A—C12A—C11A	−177.6 (6)	C15B—O2B—C12B—C11B	−178.0 (6)
C10A—C11A—C12A—O2A	−177.3 (6)	C10B—C11B—C12B—O2B	179.9 (7)
C10A—C11A—C12A—C13A	0.5 (11)	C10B—C11B—C12B—C13B	−0.6 (11)
O2A—C12A—C13A—C14A	178.2 (6)	O2B—C12B—C13B—C14B	179.9 (6)
C11A—C12A—C13A—C14A	0.5 (10)	C11B—C12B—C13B—C14B	0.5 (10)
C12A—C13A—C14A—C9A	−2.6 (10)	N2B—C9B—C14B—C13B	178.5 (6)
C12A—C13A—C14A—S1A	−177.6 (6)	C10B—C9B—C14B—C13B	−2.2 (11)
C10A—C9A—C14A—C13A	3.8 (10)	N2B—C9B—C14B—S1B	0.3 (8)
N2A—C9A—C14A—C13A	−178.3 (6)	C10B—C9B—C14B—S1B	179.6 (6)
C10A—C9A—C14A—S1A	179.6 (6)	C12B—C13B—C14B—C9B	0.9 (10)
N2A—C9A—C14A—S1A	−2.4 (8)	C12B—C13B—C14B—S1B	178.8 (5)
C8A—S1A—C14A—C13A	176.1 (7)	C8B—S1B—C14B—C9B	−0.4 (5)
C8A—S1A—C14A—C9A	0.7 (5)	C8B—S1B—C14B—C13B	−178.5 (6)

### Hydrogen-bond geometry (Å, º)

**Table:**

*D*—H···*A*	*D*—H	H···*A*	*D*···*A*	*D*—H···*A*
O1*A*—H1*A*···N1*A*	0.84	1.93	2.647 (9)	143
C13*A*—H13*A*···O1*B*	0.95	2.48	3.289 (9)	144
C15*A*—H15*A*···N2*B*^i^	0.98	2.57	3.525 (10)	166
O1*B*—H1*B*···N1*B*	0.84	1.89	2.636 (9)	147
C13*B*—H13*B*···O1*A*	0.95	2.53	3.356 (10)	145

Symmetry code: (i) −*x*+3/2, *y*−3/2, *z*−1/2.
